# Persistent Post-dural Puncture Headaches One Year After Lumbar Puncture: A Case Report

**DOI:** 10.7759/cureus.74573

**Published:** 2024-11-27

**Authors:** Zamanali Khakhar, Maeheq Mir, Soraiya Manji, Sheila Waa, Sayed K Ali

**Affiliations:** 1 School of Medicine, University of Nairobi, Nairobi, KEN; 2 School of Medicine, Royal College of Surgeons in Ireland, Dublin, IRL; 3 Department of Internal Medicine, Aga Khan University Hospital, Nairobi, KEN; 4 Department of Radiology, Aga Khan University Hospital, Nairobi, KEN

**Keywords:** cerebrospinal fluid leakage, computed tomographic myelography, epidural blood patch, headache, lumbar puncture, post-dural puncture headache, spontaneous intracranial hypotension

## Abstract

Post-dural puncture headaches usually occur when the cerebrospinal fluid (CSF) leaks due to trauma to the dura mater. This often results in spontaneous intracranial hypotension characterized by orthostatic headaches, neck stiffness, and nausea. In this case report, we discuss a 20-year-old male patient who developed symptoms of intracranial hypotension one year following a lumbar puncture. A spinal magnetic resonance imaging (MRI) revealed a CSF collection in the epidural space. This was treated successfully by an epidural blood patch (EBP) which resolved the patient's symptoms. This case highlights the necessity of recognizing delayed lumbar puncture complications and the need for a thorough history and physical exam in patients with such symptoms.

## Introduction

Cerebrospinal fluid (CSF) is a clear and colorless liquid primarily composed of water, glucose, electrolytes, and minimal amounts of protein, with albumin being the predominant protein. It is produced by the choroid plexus within the brain ventricles and circulates through the ventricular system and subarachnoid space [1]. CSF serves several functions, including protecting the brain by cushioning it from impact and providing buoyancy, which reduces pressure on the surrounding cerebral vasculature and parenchyma [2]. 

CSF leaks occur due to a tear or defect in the dura mater, the outermost layer of the meninges which serves to protect and encases the brain and spinal cord. This results in CSF leaking out of the subarachnoid space, potentially leading to spontaneous intracranial hypotension, which is characterized by a decrease in CSF pressure. The presenting symptoms include orthostatic headaches, neck stiffness, nausea, and neurological deficits due to diminished cushioning of the brain and subsequent downward displacement [2]. 

Iatrogenic causes account for a relatively small proportion of CSF leaks, with the majority due to trauma. Numerous studies have reported varying incidence rates of CSF leakage (1%-30%) following lumbar punctures [3], highlighting a significant variability. Therefore, these differences suggest that the technique and instruments employed, along with distinct individual characteristics, contribute appreciably in determining the risk of CSF leakage following a lumbar puncture [4]. Repeated lumbar punctures, suboptimal techniques, use of larger needles, and traumatic needle designs have been linked to a heightened risk of durotomies that leads to CSF leakage [5]. Reports on substantially chronic CSF leakage are limited, with very few documented cases. This case is particularly noteworthy for its unusual presentation, as CSF leakage persisted over an extended period of one year. To the best of our knowledge, the documented cases of prolonged CSF leakage identified in our review of literature were reported by Wilton et al. and Göksel et al., both of whom described cases associated with a prolonged post-dural puncture headache for 19 months and 10 years, respectively [6,7].

In this report, we seek to explicate the case of a 20-year-old male who developed chronic CSF leakage over a period of approximately one year following a lumbar puncture for spinal anesthesia administered during a hemorrhoidectomy, presenting with characteristic symptoms of intracranial hypotension. The peculiarity of this case is manifested by the long-standing, low-pressure leakage of CSF, which persisted for virtually one year and the importance of a focused history and physical exam in patients with recurrent symptoms.

## Case presentation

A 20-year-old male patient of African descent, presented to the hospital for treatment of acute gastroenteritis. He also reported a history of headaches that began one year ago following spinal anesthesia administered during a hemorrhoidectomy at a different health facility. The headaches occurred 2-3 days per week, with each episode lasting several hours. He described them as global with greater intensity at the vertex, insidious in onset, and pulsatile. The headaches were exacerbated when transitioning from a recumbent to a sitting position and were alleviated by lying down. His headaches were further aggravated by stress and prolonged sitting in the classroom. He noted that adequate hydration or consumption of coffee provided some relief from the symptoms. He denied any photophobia, phonophobia, nausea, or vomiting associated with the headache. On more severe occasions, the pain was relieved by taking paracetamol or nonsteroidal anti-inflammatory drugs (NSAIDs). He also denied any trauma to his back or head during the preceding year. There was no history suggestive of steroid use, connective tissue disorders, meningitis, neurosurgical procedures, or other factors associated with dural weakness.

Prior to his surgery last year, the patient had no history of similar headaches. He denied experiencing fever, night sweats, or weight loss. He had no other systemic symptoms except for two days of diarrhea and vomiting which was attributed to food poisoning and exacerbated his headaches. The patient had no other significant past medical history. He reported no history of smoking, alcohol consumption, or use of recreational or illicit substances. There was no significant family history of concern. He has sought care for his persistent headaches as an outpatient at various healthcare centers, multiple times, and was treated conservatively with various painkillers that decreased the intensity of the headaches but never provided complete resolution. Physical examination was essentially unremarkable. His vital signs were normal. A neurological exam revealed no cranial nerve, motor, sensory, or cerebellar deficits as well as negative Brudzinski's and Kernig's signs. There were no signs to suggest cord compression in this patient. Fundoscopy was normal with no papilledema. Blood samples were normal including the absence of signs of infection.

**Figure 1:**
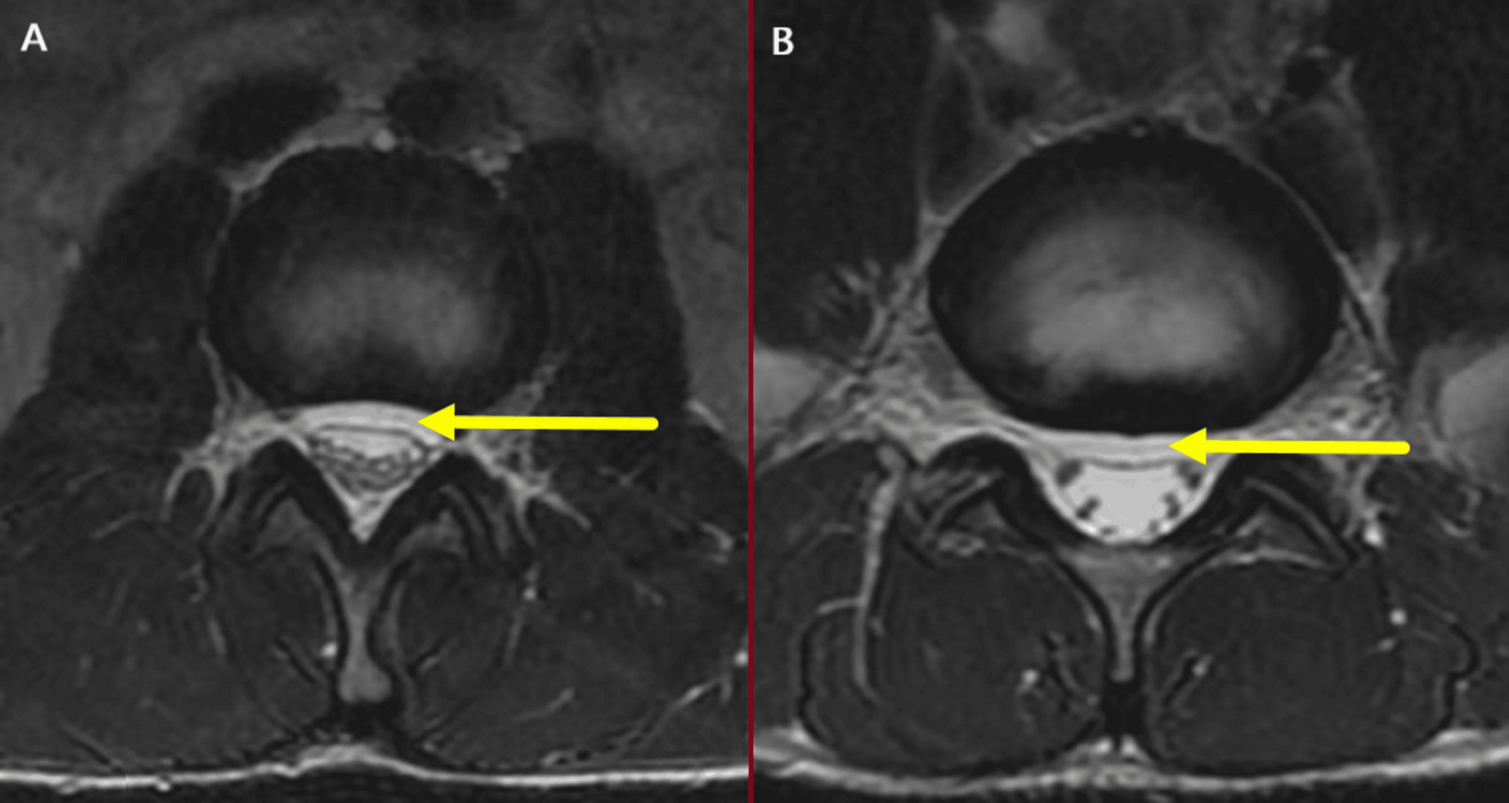
(A-B) Axial T2 images at the L2-L3 and L5-S1 levels demonstrating the ventral epidural CSF collection (yellow arrows) CSF: cerebrospinal fluid

**Figure 2:**
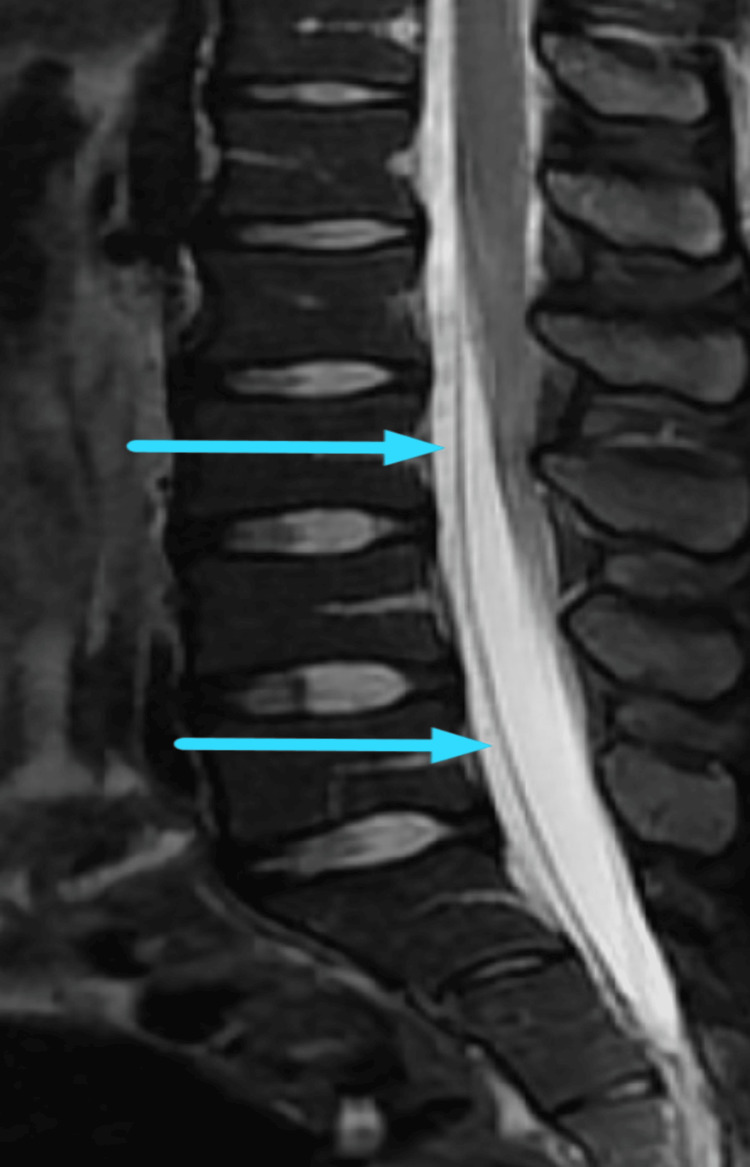
Sagittal T2 fat-suppressed image demonstrating ventral epidural CSF collection (blue arrows) CSF: cerebrospinal fluid

He was managed for acute gastroenteritis with intravenous fluids and antiemetics. His complete blood count, renal function tests, and thyroid function tests were within normal limits. Given his history suggestive of a low-pressure headache, an MRI of the brain and spine was performed. The brain MRI did not reveal any findings characteristic of CSF leakage with associated intracranial hypotension, such as diffuse pachymeningeal enhancement, reduced ventricular size, venous sinus engorgement, or subdural fluid collections. However, the spinal MRI revealed a 4 mm thick lumbar ventral epidural CSF collection (Figures 1-2). This CSF leak was thought to be a consequence of a dural puncture during a prior event of spinal anesthesia. The patient was advised on an epidural blood patch (EBP) which was successfully performed by an anesthesiologist. The EBP procedure was performed at the L4-L5 level using 10 mL of autologous blood [1]. The patient's symptoms showed significant improvement within a week following the EBP. No complications were reported post-procedure. A follow-up spinal MRI was not performed due to the patient's financial constraints.

## Discussion

CSF leak is a recognized complication associated with lumbar punctures and various neurosurgical procedures. It is therefore crucial to comprehensively understand the potential complications of performing lumbar punctures as well as the possible sequelae of a CSF leak. The delayed or even the absence of closure of the dural defect explains the prolonged leakage of CSF following durotomies. This case documents the presence of a prolonged leakage of CSF into the epidural space following a lumbar puncture, (details of the nature and technique of which are unknown), resulting in a chronic and debilitating post-lumbar puncture headache with hallmark symptoms of intracranial hypotension. The significance of this case report is underscored by the rarity of a prolonged CSF leakage and post-dural puncture headache following a lumbar puncture. The current literature references only a few cases involving extended durations of post-dural puncture headaches, notably, one lasting 19 months and another persisting for 10 years [6,7]. This report seeks to contribute to the limited body of knowledge on this unusual complication, highlighting the critical importance of recognizing and appropriately managing a persistently prolonged CSF leak, which arises from an otherwise routine procedure such as a lumbar puncture.

Post-lumbar puncture headache is the most common complication that presents following a lumbar puncture. Other serious complications include pseudomeningocele, nerve root entrapment, potential paralysis, and intracranial hemorrhage [8,9]. Leakage of CSF can be detrimental; the loss of the cushioning effect provided by the CSF increases the vulnerability of the brain parenchyma to direct trauma and further increases the risk of intracranial hemorrhage [1]. A previous case documented an event of cervical spinal cord displacement with subsequent nerve root entrapment in a young female patient, as a result of substantial accumulation of the CSF in the epidural space, due to a dural tear from diagnostic lumbar puncture [10]. 

The development of chronic CSF leakage, often presenting with symptoms such as orthostatic headache, can frequently be attributed to iatrogenic factors. These include needle gauge/size, which influences the size of the dural tear; the orientation of needle bevel; needle design, whether atraumatic or traumatic; reinsertion of the stylet prior to needle withdrawal; and the number of lumbar puncture attempts [5]. As a result, improper technique and inappropriate selection and utilization of instrumentation are implicated in the occurrence of CSF leakage. The patient’s history of intermittent orthostatic headaches following spinal anesthesia for a hemorrhoidectomy raised the suspicion of intracranial hypotension secondary to CSF leakage, thus prompting further evaluation with MRI. 

In cases of CSF leakage with associated intracranial hypotension, an MRI of the brain typically reveals diffuse pachymeningeal enhancements with contrast, reduced ventricular size, caudal displacement of the cerebellar tonsil into the foramen magnum, flattening of the basal cisterns, engorgement of venous sinuses, and the presence of subdural fluid collections [11]. An MRI of the spine may demonstrate dural collapse and may allow visualization of the CSF leakage from the spinal dural defects [12]. Computed tomographic myelography (CTM) is considered the gold standard for localizing spinal CSF leaks. It can be used effectively to determine the presence and specific location of a CSF leak [13]. In our case, axial T2-weighted imaging at the L2-L3 and L5-S1 levels revealed a ventral epidural CSF collection, which aided in confirming the diagnosis. In this case, limited access to MRI due to financial and demographic constraints contributed to prolonged symptoms that might have been resolved earlier with more readily available diagnostic resources. 

To minimize the risk of post-lumbar puncture headache and CSF leakage, several measures can be implemented. Using a needle with smaller gauge or diameter creates a smaller dural tear, reducing the potential for CSF leakage [5]. Evidence suggests that the use of atraumatic needles significantly reduces the incidence of post-lumbar puncture headache. Strupp et al. demonstrated that the atraumatic Sprotte needle notably lowered the likelihood of headaches compared to the traumatic Quincke needle [14]. Minimizing the number of lumbar puncture attempts may reduce dural trauma and prevent subsequent CSF leakage [5].

EBP is the treatment of choice for patients who present with post-dural postural headaches due to a CSF leak. The procedure involves injecting 10-20 ml of autologous blood into the lumbar epidural space near the dural tear forming a seal and preventing the leakage of the CSF [1,11]. Additionally, pressure on the thecal sac increases the pressure within the subarachnoid sac and pushes the CSF upward, thus inhibiting leakage of the CSF through the dura. While EBP achieves a success rate of 56% to 98% for postural headaches, the procedure does carry certain complications. These include paresthesias, back stiffness, and local back pain with rare complications including epidural abscess and nerve damage [11]. Contraindications to treatment with EBP include coagulopathy, an active infection, spinal stenosis, and pregnancy [15].

The patient’s repeated visits to multiple clinics, coupled with the lack of appropriate treatment and persistent symptoms, highlight the importance of a thorough history and physical exam. This can also demonstrate the necessity for heightened awareness of the spectrum of disease presentations, which poses a diagnostic challenge and emphasizes the importance of developing expertise in the recognition and management of post-dural puncture headaches.

## Conclusions

CSF leakage can lead to debilitating consequences and a poor quality of life. Healthcare providers must be cognizant of this complication, as the application of optimal techniques and the use of appropriate instrumentation are crucial in reducing the likelihood of its occurrence. The role of detailed history and physical exam in any patient, especially those with complicated symptoms, cannot be overemphasized.
